# Intranasal Vaccination Affords Localization and Persistence of Antigen-Specific CD8^+^ T Lymphocytes in the Female Reproductive Tract

**DOI:** 10.3390/vaccines4010007

**Published:** 2016-03-17

**Authors:** Shailbala Singh, Kimberly S. Schluns, Guojun Yang, Scott M. Anthony, Michael A. Barry, K. Jagannadha Sastry

**Affiliations:** 1 Department of Immunology, The University of Texas M.D. Anderson Cancer Center, Houston, TX 77054, USA; ssingh1@mdanderson.org (S.S.); kschluns@mdanderson.org (K.S.S.); gyang3@mdanderson.org (G.Y.); scottmanthony@gmail.com (S.M.A.); 2 Immunology Graduate Program, The University of Texas Graduate School of Biomedical Sciences at Houston, Houston, TX 77030, USA; 3 Department of Internal Medicine, Mayo Clinic, Rochester, MN 55905, USA; Barry.Michael@mayo.edu; 4 Department of Molecular Medicine, Mayo Clinic, Rochester, MN 55905, USA; 5 Division of Infectious Diseases, Mayo Clinic, Rochester, MN 55905, USA; 6 Translational Immunovirology Program, Mayo Clinic, Rochester, MN 55905, USA; 7 Department of Immunology, Mayo Clinic, Rochester, MN 55905, USA

**Keywords:** Female reproductive tract, CD8^+^ T cells, intranasal immunization

## Abstract

Immunization strategies generating large numbers of antigen-specific T cells in the female reproductive tract (FRT) can provide barrier protection against sexually-transmitted pathogens, such as the human immunodeficiency virus (HIV) and human papillomaviruses (HPV). The kinetics and mechanisms of regulation of vaccine-induced adaptive T cell-mediated immune responses in FRT are less well defined. We present here evidence for intranasal delivery of the model antigen ovalbumin (OVA) along with alpha-galactosylceramide adjuvant as a protein vaccine to induce significantly higher levels of antigen-specific effector and memory CD8^+^ T cells in the FRT, relative to other systemic and mucosal tissues. Antibody blocking of the CXCR3 receptor significantly reduced antigen-specific CD8^+^ T cells subsequent to intranasal delivery of the protein vaccine suggesting an important role for the CXCR3 chemokine-receptor signaling for T cell trafficking. Further, intranasal vaccination with an adenoviral vector expressing OVA or HIV-1 envelope was as effective as intramuscular vaccination for generating OVA- or ENV-specific immunity in the FRT. These results support the application of the needle-free intranasal route as a practical approach to delivering protein as well as DNA/virus vector-based vaccines for efficient induction of effector and memory T cell immunity in the FRT.

## 1. Introduction

Since the majority of pathogenic infections are initiated at the mucosal surfaces, induction of robust immunity at these sites is critical for protection [1,2,3,4,5,6]. For sexually-transmitted pathogens, such as the human immunodeficiency virus (HIV) and human papilloma virus (HPV), generation of antigen-specific cell-mediated immunity in the female reproductive tract (FRT) is essential so that CD8^+^ T cell responses can prevent the establishment of infection [2,7].

The highly-compartmentalized nature of the mucosal immune system enables the generation of immune responses at the site of immunization as well as distant mucosal sites [8,9]. Intranasal (IN) immunization has been shown to effectively induce IgA and IgG responses in the FRT at levels comparable to those after vaginal immunization and studies with herpes simplex virus-2 (HSV-2) and *Chlamydia trachomatis* have also demonstrated induction of antigen-specific CD4^+^ T cell responses in the genital mucosa [5,6]. However, the effective route for vaccine-mediated induction of CD8 T cell responses in the FRT is less well established [8,10,11,12]. Studies also suggest that mucosal specific integrins, such as α4β7, and chemokine receptors, such as CCR9, CR10, and CXCR3, expressed in the mucosal microenvironment could be involved in achieving broadly-disseminated mucosal immunity but the chemokine signaling mechanisms that direct the vaccine-induced T cell responses to FRT remain poorly defined [13,14,15,16]. To develop effective vaccines against sexually-transmitted infections, an understanding of the phenotype and kinetics of development and persistence of the CD8^+^ T cell immune response in FRT is essential.

Using peptide or protein antigens specific to HIV and HPV, along with alpha-galactosylceramide (αGalCer) as an adjuvant, we and the others have previously reported that mucosal IN and oral/sublingual immunizations, can induce broadly disseminated antigen-specific antibodies and cell-mediated immune responses [17,18,19,20,21]. In the current study, we investigate the effectiveness of IN vaccination at inducing persistent CD8^+^ T cell mediated immunity specifically in the FRT. The study also tested the potential role for CXCR3 mediated signaling in localizing CD8^+^ T cell immunity to the FRT. Data from these studies show the IN route is effective in generating persistent antigen-specific CD8^+^ T cells in the FRT supporting IN delivery as a practical and needle-free strategy for mass-scale vaccination campaigns to induce protective barrier immunity.

## 2. Materials and Methods

### 2.1. Animals

Female C57BL/6 and C57BL/6 X BALB/c (CB6F1) mice at six weeks of age were purchased from the National Cancer Institute (Frederick, MD, USA) and maintained in specific pathogen-free environment at the institutional animal facility. The animal facility is fully accredited by the Association for Assessment and Accreditation of Laboratory Animals Care International. All animal procedures were conducted in compliance with the institutionally-approved protocols.

### 2.2. Reagents

The endotoxin-free ovalbumin (OVA) protein was purchased from InvivoGen (San Diego, CA, USA). The alpha-galactosylceramide (αGalCer) was purchased from Diagnocine LLC (Hackensack, NJ, USA) and dissolved in dimethyl sulfoxide, (Sigma, St. Louis, MO, USA) at a stock concentration of 1 mg/mL. APC-labeled H-2Db restricted OVA cytotoxic T lymphocyte (CTL) epitope SIINFEKL and H-2Dk restricted HIV envelope CTL epitope IGPGRAFYA containing tetramers were procured from the MHC tetramer production facility at Baylor College of Medicine (Houston, TX, USA) and was used for the detection and analysis of peptide-specific CD8^+^ T cells in different tissues by flow cytometry. Adenoviral vectors expressing ovalbumin (Ad-OVA) and HIV-1 envelope (Ad-ENV) were prepared according to previously described protocols [22].

### 2.3. Adoptive Transfer of OT-I Cells and In Vivo Blocking of CXCR3

For adoptive transfer of OT-I, lymph nodes were collected from untreated Rag2−/− OT-I mice (CD45.1^+^) and 1 × 10^6^ cells were transferred to congenic C57BL/6 mice (CD45.2^+^). The animals were immunized by IN route one day after adoptive transfer of cells. For blocking of CXCR3 on lymphocytes, mice were treated with anti-CXCR3 monoclonal abs (MAbs) (intraperitoneal injections of 200 µg) one day before and one day after immunization.

### 2.4. Immunizations

For IN immunization, mice were first anesthetized with ketamine and xylazine hydrochloride (100 mg/kg and 10 mg/kg respectively, i.p.) and then each animal received an administration of 100 µg of OVA protein, either alone or with 2 µg of αGalCer in 15–20 uL volume as previously described [17,18]. These doses of antigen and adjuvant were chosen based on our past studies and literature reports [15,16]. Replication defective adenoviral vectors were administered at a dose of 1 × 10^10^ viral particles (vp). For intramuscular immunizations, the inoculum was injected in the caudal thigh muscle using a 27 G, 0.5 inch tuberculin syringe.

### 2.5. Analyses of the Phenotype of Antigen Specific T Lymphocytes in FRT

Antigen-specific T cell responses were determined at different time points post-immunization. Tissues were harvested six and 58 days post-immunization. For analysis of circulating T cells, blood was collected by retro-orbital route. For isolation of lymphocytes from FRT and intestines, previously described protocols were used [23,24]. Briefly, lymphocytes from the FRT (vagina, uterus, and uterine horns) were isolated by incubating the tissue with EDTA (5 mM) for one hour, followed by digestion with collagenase (15,000 units/mL) for one hour at 37 °C and then the purified lymphocyte population was recovered by discontinuous Percoll gradient centrifugation. The presence and phenotype of antigen-specific CD8^+^ T lymphocytes in the FRT, spleen, draining cervical lymph nodes, and intestinal lamina propria (LPL) and intra-epithelial (IEL) lymphocytes was determined by flow cytometry. Lymphocytes were stained with either APC-conjugated MHC-I tetramer complexed with OVA peptide or HIV envelope peptide, pacific blue-conjugated anti-CD44 (clone IM7 BD Biosciences, San Jose, CA, USA), PerCP Cy5.5 conjugated anti-CD8α (clone 53-6.7 BD Biosciences, San Jose, CA, USA), PE conjugated anti-CD8β (clone H35-17.2 BD Biosciences, San Jose, CA, USA), and FITC-conjugated anti-CD62L (clone MEL-14 BD Biosciences, San Jose, CA, USA) antibodies. Percentage of CD44^hi^, tetramer positive cells among total CD8^+^ lymphocytes was determined for animals receiving immunization with either OVA alone or OVA + αGalCer. Flow cytometric data were acquired with a LSRII (BD Biosciences) or LSR Fortessa (BD Biosciences, San Jose, CA, USA) and analyzed with Flowjo software version 9.7.6 (Flowjo LLC, Ashland, OR, USA).

### 2.6. Statistical Analysis

The immune responses were expressed as averages of 3–6 animals/group. Non-parametric test (Mann-Whitney test) was used to determine the significance of difference between different immunization groups. All analyses were performed using GraphPad Prism (version 6) and *p* ≤ 0.05 was considered statistically significant.

## 3. Results and Discussion

### 3.1. Intranasal Immunization Preferentially Induces Antigen-Specific CD8^+^ T Lymphocytes in the FRT

**Figure 1:**
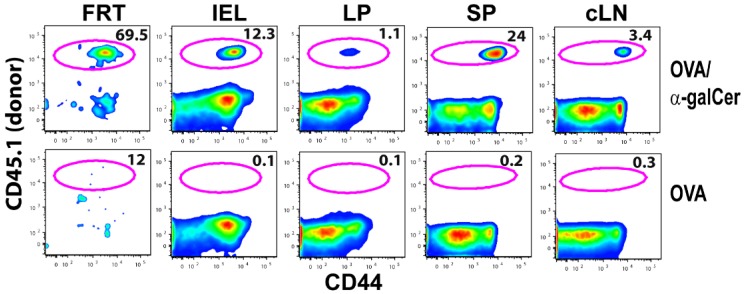
IN vaccination preferentially induces OVA specific CD8^+^ T cells in FRT. Plots show frequency of OT-I T cells among CD8^+^ T cells present in FRT, intestinal intraepithelial lymphocytes (IEL), lamina propria (LP),spleen (SP), and cervical LN(cLN) six days after IN vaccination with either OVA plus αGalCer or OVA alone.

We used the adoptive transfer of ovalbumin (OVA)-specific OT-I TCR transgenic (Tg) CD8^+^ T cells as a model system to explore the kinetics of antigen-specific T cells in the FRT of the mice following IN immunization. CD45.1^+^ naive OT-I cells (0.5 × 10^6^ cells) were adoptively transferred to congenic C57Bl/6 mice (CD45.2^+^) and the recipient animals were immunized with either OVA + αGalCer or OVA alone by IN route one day later. Six days post immunization, lymphocytes from the FRT (vagina, uterus, and uterine horns), intestinal epithelium, and lamina propria of the small intestines, spleen, and cervical lymph nodes were isolated and analyzed by flow cytometry. Donor OT-I T cells were identified after gating on live CD45.1^+^CD8α^+^CD8β^+^. Inclusion of CD8β for FRT and intestinal lymphocytes enabled identification of conventional CD8α^+^β^+^ T cells from unconventional CD8αα^+^ T cells, which are often present in mucosal tissues. Surprisingly, among mice immunized with OVA + αGalCer, a majority of CD8^+^ T cells in the FRT were OT-I T cells (Figure 1). This is in striking contrast to the frequency of OT-I T cells present in the intestinal IEL or lamina propria, or the LNs and spleen. Overall, these data suggest that IN vaccination more specifically directs T cell responses to the reproductive tract than other mucosal tissues.

In a different experiment, persistence of CD8^+^ T cells after vaccination in both systemic and mucosal tissues of mice was evaluated by comparing the frequencies of OT-I cells on days six and 58 post-immunization. Inclusion of αGalCer as an adjuvant with OVA induced higher frequencies of activated OVA-specific CD8^+^ T cells (CD45.1^+^CD44^hi^CD8^+^) in blood and spleen (Table 1).

**Figure 2:**
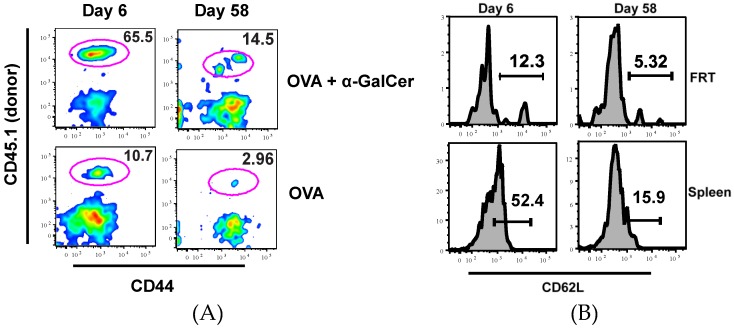
IN vaccination induces persistent OVA specific CD8^+^ T cells in FRT. Plots show frequency of OT-I T cells among CD8^+^ T cells present in FRT of mice immunized with OVA or OVA + αGalCer at six and 58 days after IN vaccination (**A**). Plot shows CD62L expression on OT-I cells isolated from the FRT and spleen of mice at different times after immunization with OVA + αGalCer (**B**).

Similarly, a greater population of antigen specific CD8^+^ T cells was induced in the FRT of mice immunized with OVA + αGalCer. More importantly, OT-I T cells were still present in the FRT eight weeks after vaccination (Figure 2A and Table 2) demonstrating IN vaccination with protein/adjuvant is sufficient in inducing memory T cells in the FRT. At both time points, OT-I T cells in the FRT were all CD44^hi^ and CD62L^lo^ (~90%–100%), consistent with the Tem/Trm phenotype (Figure 2) [25,26].

### 3.2. Potential Role of CXCR3 Signaling for Intranasal Induction of Antigen-Specific CD8^+^ T Cells Migration into FRT

**Figure 3:**
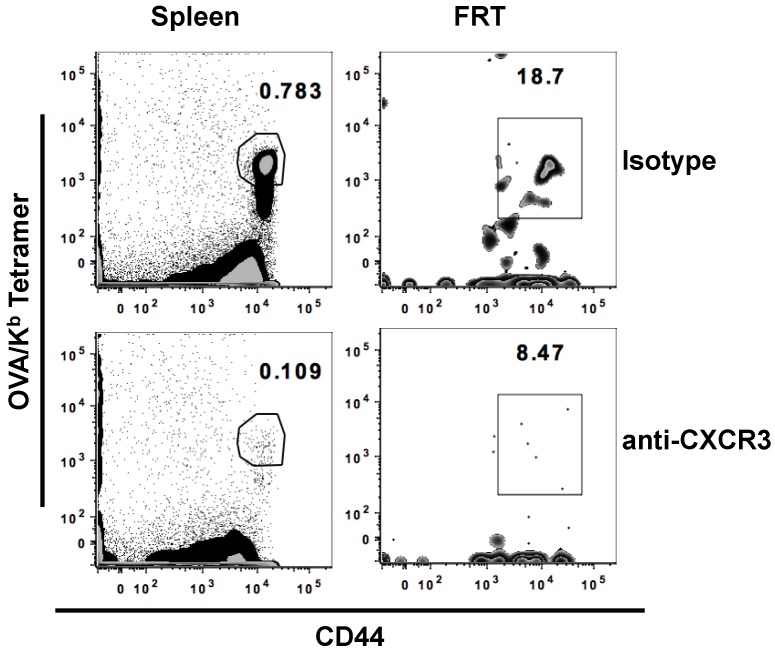
CXCR3 blocking limits the generation of antigen-specific T cells. Separate groups of mice were treated with either CXCR3 blocking antibody or isotype antibody a day before and a day after IN immunization with OVA and αGalCer. Mice were sacrificed on day 7 post-immunization and lymphocytes were isolated from the female reproductive tract (FRT) and spleen. Representative scatterplots of activated antigen specific CD8^+^ T cells in the different tissues is presented.

Entry and localization of T cells from the blood into tissues is regulated by interaction between chemokine receptors on the surface of T cells and their specific ligands in different tissues. Studies have shown that CXCR3, a chemokine receptor expressed on effector T cells, along with its ligands, plays a critical function in the trafficking of CD8^+^ T cells to infected mucosal tissues, such as lung and FRT; however, the involvement of CXCR3 in a vaccine setting where the FRT is not encountering immune activation is unknown [27,28,29,30,31]. Therefore, to determine the role of CXCR3 in generating antigen-specific CD8^+^ T cells in the FRT after IN immunization with OVA and αGalCer, mice were administered α-CXCR3 (blocking) antibody a day before and a day after immunization. Mice were sacrificed seven days post-immunization and the immune responses were analyzed in the spleen, and FRT. With IN immunization, the frequency of antigen-specific CD8^+^ T cells present in both mucosal (FRT) and systemic (spleen) tissues was significantly reduced with the blocking of CXCR3 function (Figure 3 and Table 3). This observation provides the first indication for a potential role of CXCR3 signaling after IN vaccination on the induction of antigen specific T cells in multiple tissues including the draining cervical lymph nodes and the FRT, suggesting that the blocking of CXCR3 inhibits the response at the proximal as well as distant locations.

### 3.3. IN Route is Effective to Deliver Both Protein and DNA/Virus-Vector Based Vaccines for Efficient Generation and Localization of Antigen-Specific CD8^+^ T Cell Responses in the Female Reproductive Tract

**Figure 4:**
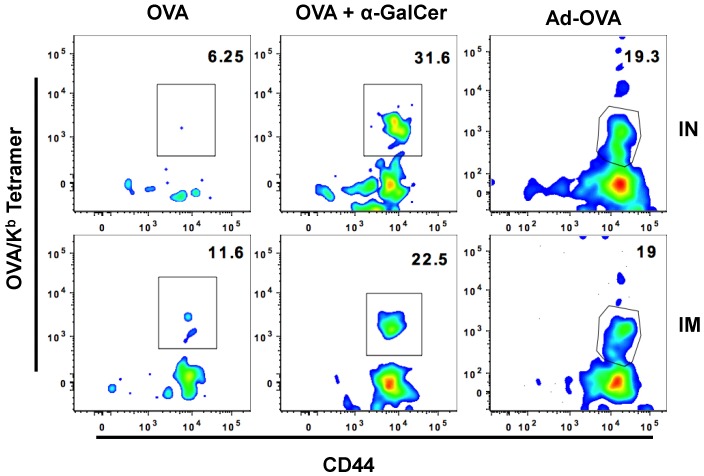
IN and IM immunization are equally effective at inducing OVA-specific CD8^+^ T cells in FRT. Plots show frequency of Ova-tetramer specific CD8^+^ T cells among CD8^+^ T cells present in FRT of mice immunized with either OVA, OVA + αGalCer, or Ad-OVA seven days after IN vaccination.

A majority of vaccinations, such as those against Hepatitis B virus and human papilloma virus that are either inactivated virus or viral proteins, are administered by systemic intramuscular route [32]. We compared the effectiveness of mucosal IN vaccination with systemic intramuscular vaccination in generating antigen-specific CD8^+^ T cells in the FRT after immunizing with either OVA protein alone, OVA protein with αGalCer, or Adenoviral vector expressing OVA (Ad-OVA). We found that the IN route was as effective as the intramuscular route in generating OVA-specific effector CD8^+^ T cells in the FRT seven days post-immunization (Figure 4 and Table 4).

**Figure 5:**
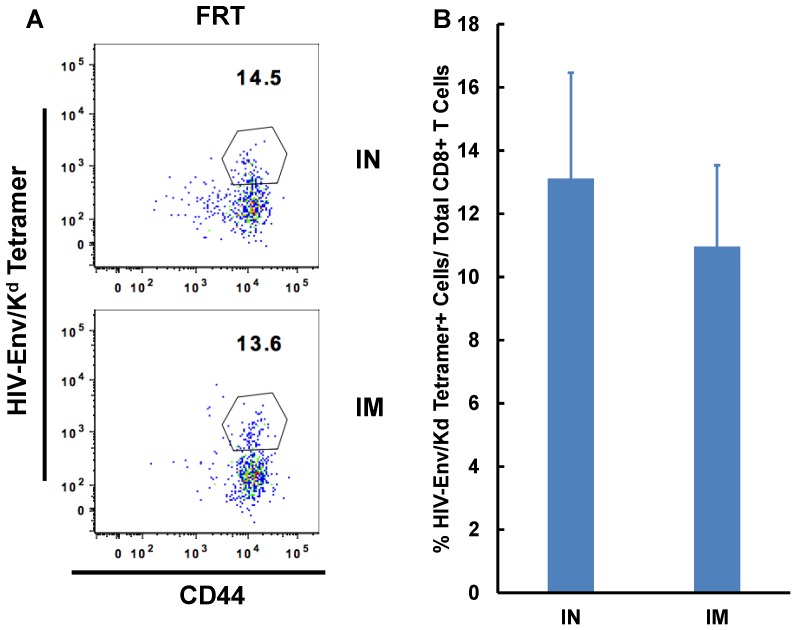
Intranasal immunization is as effective as intramuscular immunization at inducing HIV envelope-specific CD8^+^ T cell immunity in the FRT. Separate groups of mice (n = 3–6) were immunized by either IN route or intramuscular route with Adenoviral vector expressing HIV-1 envelope (Ad-Env). Mice were sacrificed on day seven post-immunization and lymphocytes were isolated from the female reproductive tract (FRT) and spleen. Localization and activation of antigen-specific CD8^+^ T cells in the different tissues was determined by flow cytometry. Representative scatterplot of activated antigen-specific CD8^+^ T cells cells in the different immunization groups is presented (**A**). Graphs with the average frequency of antigen-specific cells isolated from the FRT and spleen of each group are shown (**B**).

Flu mist is the only IN vaccine approved for use in humans and it generates anti-influenza virus immunity in the respiratory tract of the recipients [33]. To mimic an actual vaccination strategy against HIV, we evaluated the generation of HIV gp120 specific immune responses. C57Bl/6F1 Cr mice were immunized with adenoviral vector expressing HIV gp120 protein (Ad-ENV) either by IN or intramuscular route and generation of antigen specific effector CD8^+^ T cell immunity was determined seven days post-immunization. Similar to the observations with Ad-OVA, HIV envelope-specific responses in the FRT were similar between the two routes of immunization (Figure 5).

## 4. Discussion

Vaccines that can be delivered without the need for sterile syringes and needles will offer a simple and safe platform format for global vaccination campaigns against infectious agents, such as HIV and HPV that are sexually transmitted. Immunization strategies targeting protective antigen-specific CD8^+^ T cells responses to the female reproductive tract (FRT) are, therefore, important to generate mucosal barrier protection as well as systemic immunity [2].

Our study showed that IN route of vaccine delivery was effective at inducing antigen-specific CD8^+^ T cell responses in the FRT of immunized mice. Using the OT-I transgenic model system, we obtained evidence to show that administration of OVA protein, along with αGalCer adjuvant, as a model protein vaccine elicited a persistent CD8^+^ T cell-mediated immune response in the FRT that is characterized by CD44^hi^CD62L^lo^ expression. Importantly, IN immunization preferentially targeted CD8^+^ T cell immunity to the FRT, relative to gut associated mucosa. Using recombinant adenoviral vectors, Kaufman *et al.* have shown that with IM delivery the systemic antigen-specific CD8^+^ T cells can migrate to the mucosal surfaces specifically to the gastrointestinal compartments; however, these studies did not investigate the influence on FRT homing of CD8^+^ T cells [34]. Thus, relative to other vaccination studies in the literature delivering DNA or viral-vector immunogens by the intramuscular route that showed induction antigen-specific mucosal immune responses, our study highlights the effectiveness of the needle-free IN delivery strategy for protein-based vaccines towards induction of antigen-specific CD8^+^ T cells in the FRT [34,35,36]. Furthermore, our studies showed that the IN route is equal in its potency to the IM route for delivering protein as well as DNA or viral-vector immunogens for the localization and persistence of CD8^+^ T cell immunity in the FRT.

Most mucosal immune cells are educated at specific inductive sites in the local mucosal-associated lymphoid tissues (MALT) and, subsequently, move into and protect mucosal barriers [1,9,33]. The trafficking of antigen-induced T cells to the various mucosal sites is dependent on the involvement of different homing receptors on the cells [37,38,39] along with the primary location of T cell activation [40,41]. Our studies showed that anti-CXCR3 antibody treatment significantly reduced CD8^+^ T cell responses in the FRT, spleen, and draining lymph nodes. While it supports a potential role for this chemokine receptor CXCR3 signaling in the localization of CD8^+^ T cells in the FRT after protein vaccine immunization, it also demonstrates the relevance of CXCR3 in the induction of CD8^+^ T cell-mediated immune responses after IN immunization. These results are in line with literature reports describing genital infection with HSV-2 to be characterized by the expression of chemokines including CXCL9 and CXCL10, which interact with the chemokine receptor CXCR3, and that deficiency of these chemokines in the knockout animals rendered them to be more susceptible to infection with HSV [29,42]. Expression of CXCR3, receptor for the CXCL9 chemokine, on antigen-specific CD8^+^ T cells has been shown to have a function in directing the migration of activated T lymphocytes to mucosal tissues [29,43,44].

## 5. Conclusions

The current study highlights that IN immunization with either protein or viral-vectored vaccines has the potential to generate robust and durable antigen-specific CD8^+^ T cell immunity in the FRT that may be dependent on CXCR3-mediated signaling. The advantage of the ability to generate mucosal barrier immunity underscores the potential of IN delivery as a convenient needle-free option for immunization against sexually-transmitted pathogens.

## Figures and Tables

**Table 1:** Average frequency (± std. dev) of antigen specific CD8^+^ T cells detected in immunized mice.

	Blood		Spleen	
	OVA	OVA + αGalCer	OVA	OVA + αGalCer
**Day 6**	0.66 (± 0.41)	11.02(± 5.72)	0.26(± 0.03)	3.09 (± 1.39)
**Day 58**	0.03(± 0.01)	0.75(± 0.69)	0.001(± 0.002)	0.79(± 0.36)

**Table 2:** Average number (± std. dev) of antigen-specific CD8^+^ T cells isolated from the FRT of immunized mice.

	OVA	OVA + αGalCer
**Day 6**	4(± 3)	104(± 59)
**Day 58**	7(± 5)	42(± 14)

**Table 3:** Average frequency (± std. dev) of antigen specific CD8^+^ T cells detected in immunized mice.

	Isotype	a-CXCR3
**Spleen**	0.71 (±0.53)	0.14(±0.13) *
**DLN**	0.15(±0.02)	0.06(±0.01) *
**FRT**	15.52 (±3.22)	10.97(±2.86) *

* Statistically significant difference (*p* ≤ 0.05) in the frequency of antigen specific CD8^+^ T cells between groups of mice receiving CXCR3 blocking antibody and isotype antibody; Draining lymph node (DLN) are cervical lymph nodes draining the nasal tissue.

**Table 4:** Average frequency (± std. dev) of antigen specific CD8^+^ T cells isolated from the FRT of immunized mice.

	IN	IM
**OVA**	0	5.412(± 6.01)
**OVA + αGalCer**	16.45(± 9.98)	19.34(± 17.01)
**Ad-OVA**	30.66 (± 8.93)	33.95(± 8.54)

## Abbreviations

The following abbreviations were used in the manuscript:

FRT: Female Reproductive Tract
IN: Intranasal
IM: Intramuscular
OVA: Ovalbumin
ENV: HIV envelope
Ad: Adenovirus vector
αGalCer: Alpha galactosylceramide
